# Bronchogenic cyst removal via thoracoscopic surgery in the prone position: A case report and literature review

**DOI:** 10.1016/j.ijscr.2019.05.064

**Published:** 2019-06-08

**Authors:** Yoshihiro Ota, Takafumi Watanabe, Kosuke Takahashi, Takeshi Suda, Shingo Tachibana, Jun Matsubayashi, Yuichi Nagakawa, Yoshiaki Osaka, Kenji Katsumata, Akihiko Tsuchida

**Affiliations:** a Department of Gastrointestinal and Pediatric Surgery, Tokyo Medical University, Tokyo, Japan; b Department of Anatomic Pathology, Tokyo Medical University, Tokyo, Japan

**Keywords:** Bronchogenic cyst, Imaging, Thoracoscopic surgery, Prone position

## Abstract

**Introduction:**

Mediastinal bronchogenic cysts are encountered relatively often, but in many cases, diagnosis using imaging modalities, is difficult. Early surgical excision of bronchogenic cysts is recommended as a diagnostic and therapeutic measure. Here, we report the case of patient with a lower mediastinal bronchogenic cyst, who was treated using thoracoscopic surgery with prone positioning and include a review of literature on diagnosis and treatment of this condition.

**Presentation of case:**

The patient was a 66-year-old woman with an asymptomatic cystic lesion in the posterior, lower mediastinum. The lesion was diagnosed as an esophageal cyst using preoperative imaging and was scheduled for thoracoscopic removal with the patient in the prone position. Intraoperatively, the lesion was found to have no continuity with the esophageal wall and was easily separated from it. Moreover, a cord extending to the lesion, appeared to arise from the crura of the diaphragm. On histopathological examination of the extracted mass, the lesion was diagnosed as a bronchogenic cyst. Postoperatively, the patient recovered uneventfully and was discharged after 7 days.

**Conclusion:**

Thoracoscopic mediastinal cystectomy with the patient in the prone position may be an optimal surgical strategy for the treatment of bronchogenic cysts in the posterior, lower mediastinum.

## Highlights

• Bronchogenic cysts rarely arise from the diaphragm.
• Non-specific imaging features sometimes make a preoperative diagnosis difficult.
• An early diagnostic surgical excision is the most definitive method of management.
• Posterior mediastinal bronchogenic cysts can be excised thoracoscopically.
• Intraoperative prone positioning of the patient enhances the ease of this approach.

## 1 Introduction

Bronchogenic cysts are a type of early embryonic foregut cystic malformation that generally occur in the thoracic cavity and may rarely involve the diaphragm [1,2]. Bronchogenic cysts have non-specific imaging features in some cases, which makes their differential diagnosis difficult. Furthermore, these cysts clinically present with infection, bleeding, and respiratory disorders and can become malignant. Therefore, early surgical excision of bronchogenic cysts is recommended as a diagnostic treatment, to relieve clinical symptoms, and to prevent complications [[3], [4], [5]]. Here, we report the case of a patient with a bronchogenic cyst, who was easily treated via thoracoscopic surgery in the prone position and also include a literature review on this topic.

This work has been reported in line with the SCARE criteria [6].

## 2 Presentation of case

**Fig. 1:**
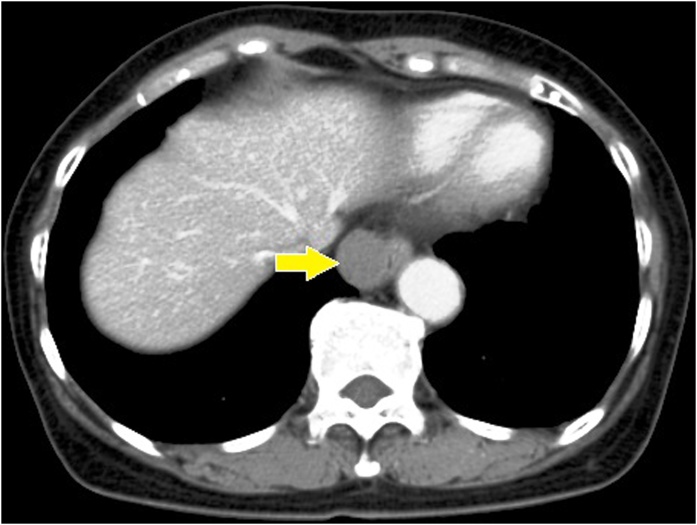
Findings from computed tomography scanning. CT showing a localized low-density area on the right side of the esophagus in the lower part of the mediastinum (arrow).

**Fig. 2:**
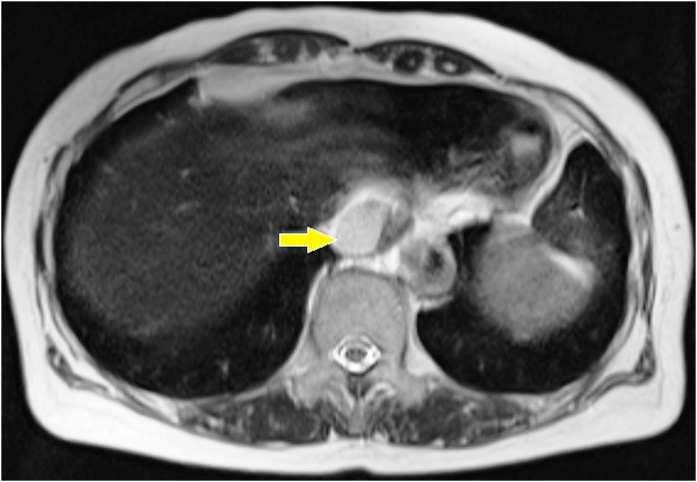
Magnetic resonance imaging findings. T2-weighted image showing a cystic mass with high signal intensity in the lower thoracic esophagus (arrow).

**Fig. 3:**
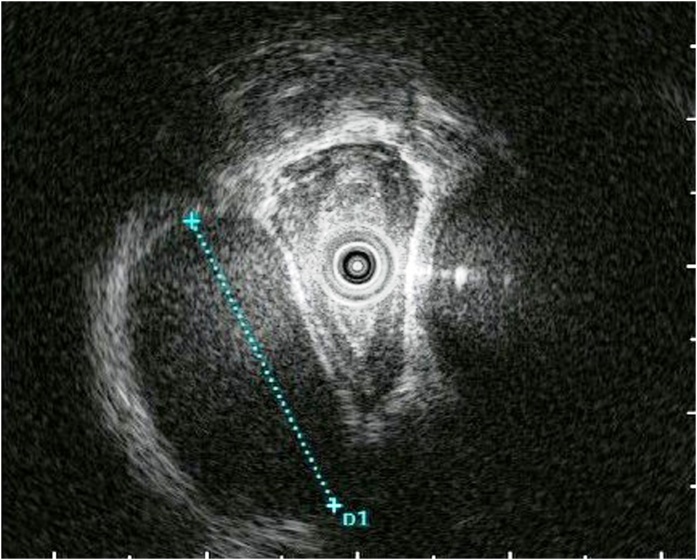
Endoscopic ultrasound findings. EUS image showing a cystic tumor in the esophageal mucosa, with no abnormality, nodules, or partitions within the cyst (dotted line).

**Fig. 4:**
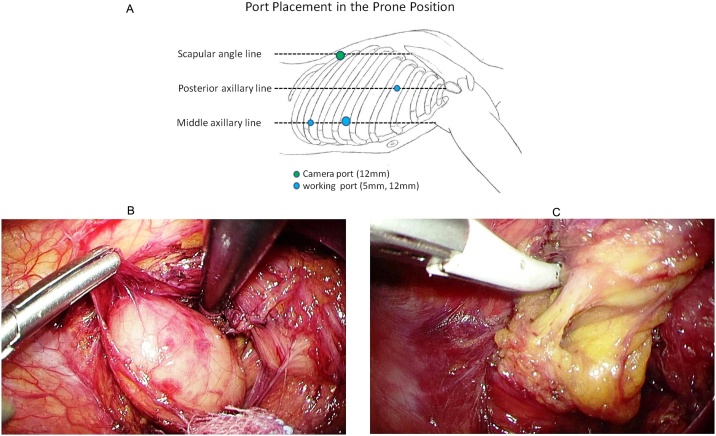
Thoracoscopic procedure and findings. A. Placement of four ports. The camera port is inserted in the 9^th^ intercostal space on the scapular line (linea scapularis), and the working ports are inserted in the 4^th^ intercostal space on the posterior axillary line and in the 6^th^ and 8^th^ intercostal spaces on the mid-axillary line. B. and C. Intraoperative findings. The lesion could be easily separated from the esophagus and is seen to be located on the crura of the diaphragm.

**Fig. 5:**
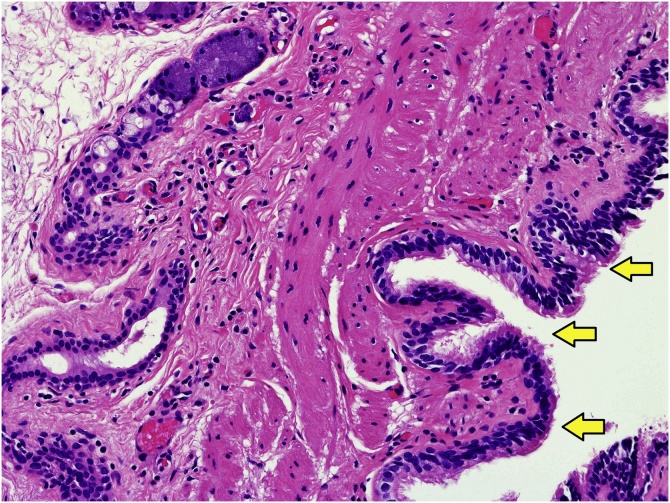
Pathological findings. The wall of the lesion is composed of fibrous fatty tissue and is lined internally with a typical pseudostratified ciliated columnar epithelium (arrow) (hematoxylin & eosin [H&E] stain, ×200).

A 66-year-old Japanese woman was observed to have a paraesophageal mass during a health checkup, for which, she was admitted to the hospital in 2016. She had undergone cholecystectomy for cholelithiasis at 30 years of age and continued to be followed-up postoperatively in the outpatient clinic, once a year. She had no family history of genetic diseases. History and examination revealed no history of postoperative adverse events following the cholecystectomy. As malignancy was suspected, tests were performed, which revealed normal levels of carcinoembryonic antigen (CEA) and carbohydrate antigen 19-9 (CA19-9) at 0.8 ng/mL and 4.8 pg/mL, respectively. Chest computed tomography (CT) showed a localized area of low density, measuring approximately 2.8 × 2.5 × 2.3 cm^3^, on the right side of the esophagus in the lower part of the mediastinum (Fig. 1). T2-weighted magnetic resonance imaging (MRI) showed a cystic and smooth-edged mass with high signal intensity in the lower thoracic esophagus (Fig. 2). Endoscopic ultrasound revealed a cystic tumor of the esophageal mucosa with no abnormality, nodule, or partitions within (Fig. 3). Therefore, we initially believed that the lesion was an esophageal cyst and planned for its removal via thoracoscopic surgery. Since we usually perform thoracoscopic esophagectomy for esophageal carcinoma with the patient in the prone position, the same method was adopted for this case. Four chest trocars were introduced (Fig. 4A). During the surgery, the lesion could be easily separated from the esophagus and was seen to be located on the crura of the diaphragm rather than on the esophagus (Fig. 4B and C). The surface of the mass was smooth and complete. Pathological examination revealed that the lesion contained fibrous and fatty wall-structures and was internally lined with typical pseudostratified ciliated columnar epithelium (Fig. 5). Thus, the lesion was diagnosed as a bronchogenic cyst arising from the diaphragm. Postoperatively, the patient recovered uneventfully and was discharged from the hospital after 7 days. No recurrence was noted during the 2-year follow-up.

## 3 Discussion

Bronchogenic cysts are considered to be caused by an aberrant germination or the abandonment of a tracheobronchial rudiment originating from the ventral lung bud of the foregut, during the embryonic period [7]. Most bronchogenic cysts occur in the lungs and mediastinum and rarely arise from the diaphragm [1,2]. The site of occurrence of bronchogenic cysts depends on their timing of development during embryogenesis. Mediastinal bronchogenic cysts are classified into paratracheal, carinal, paraesophageal, hilar, and miscellaneous subtypes [8]. We believe that because the diaphragm has not yet developed at the time of generation of the respiratory primordium from the foregut, bronchial cysts arise as malformed thoracoperitoneal ducts. This is supported by cases showing dumbbell-shaped cysts extending through the diaphragm, arising from the abdominal cavity and the diaphragm and straddling both abdominal and thoracic cavities [9].

While chest CT examination clarifies the relationship between the cyst location and the surrounding organs, its utility in identifying the type of cyst varies, depending on the nature of the cyst contents. Hence, the usefulness of CT in the preoperative diagnosis of bronchogenic cysts is limited, with a diagnostic rate of 60–70% [3]. The differential diagnoses of pulmonary bronchogenic cysts include tumor, pulmonary fraction, infectious cyst etc., and those of the mediastinal type include neurogenic tumor, teratoma, lymphoma, pericardial cyst, esophageal cyst, lymph node enlargement etc. A comparative study [4] on the diagnostic ability of CT and MRI examination for diagnosis of bronchogenic cysts showed that the diagnostic rate of CT was 69.2%, while that of MRI was 100%, because of the characteristic high signal intensity of the lesion in T2-weighted images.

The typical histopathological diagnostic feature of bronchogenic cysts is the presence of ciliated columnar epithelial cells along the inner surface of the cyst. The inner cyst wall may also contain cartilage and smooth muscle components, though these are not essential for diagnosis. In this case, the cyst was covered with multi-rowed ciliated epithelium on the inner surface, the cyst walls were surrounded by smooth muscles of variable thickness, and glandular tissue (a fascicle of nerve fibers and bronchial glands) was also observed, aiding in the diagnosis.

In adults, the treatment for suspected bronchogenic cysts is surgical removal. Early surgical excision is preferred because, making a definitive preoperative diagnosis is difficult in some cases, the risk of surgery is higher after the appearance of symptoms, and parts of the cyst have been reported to be malignant in previous studies [[2], [3], [4], [5]]. Some studies have suggested a conservative follow-up of asymptomatic patients with suspected bronchogenic cysts. However, we believe that an early resection is desirable, if it can be performed safely using a minimally invasive technique like thoracoscopic surgery. Although, there have been reports of cases with recurrence, following an incomplete resection [10,11]. As a complete resection of the cyst is essential to prevent recurrence, intraoperative transition to open thoracotomy should be implemented for cysts with dense inflammatory adhesions with the surrounding organs, which can make a complete thoracoscopic resection difficult.

Most thoracoscopic mediastinal cystectomies are performed with the patient in the lateral position. However, in this case, we performed thoracoscopic surgery with the patient in the prone position, as is our practice for esophageal cancer resection. The reasons for positioning the patient in this manner included a preoperative misdiagnosis as an esophageal cyst, a greater familiarity with this positioning as it is regularly used by us for esophageal cancer patients, and the location of the lesion in the posterior and lower mediastinum, which provided us with a good visual field on prone positioning, as the lungs moved ventrally due to gravitational effect. Some studies have reported that surgery in the prone position is not as beneficial as that in the semi-prone position because lung contraction due to gravity is better with the latter positioning, and it is easier to transition to open thoracotomy, if required [12,13]. However, Palanivelu et al. [14,15] reported that in the prone position, the lung was pushed ventrally during a minimally invasive esophagectomy, creating an artificial pneumothorax (pressure: 6–8 mmHg), thus providing a good visual field, allowing safe surgery, and resulting in less postoperative complications than those occurring in a surgery performed using the left lateral position. Moreover, using this method, the monitor is placed on the left side of the patient, so that the operator and assistant standing on the right side of the patient can both use the same screen. It also allows visualization of the mediastinum in the frontal view and maintenance of good hand-eye coordination while navigating the entire mediastinum, although it is not considered suitable for anterior mediastinal lesions [13]. Fabian et al. [16,17] reported that, compared to lateral positioning, in a prone patient, the operator’s forearm fatigue is minimized because the wrist is maintained in a neutral position. Furthermore, the operative duration is lower and the learning curve moderate, when thoracoscopic surgery is performed using prone positioning. With its advantages of good operability and preventing accumulation of blood and exudates in the operative field, prone positioning of the patient is more suitable for tumor excision without lymph node dissection, as in this case. However, this approach should be selected based on the patient’s general condition, the tumor location, and the skill of the operator. Particularly, in obese patients or in those with chronic obstructive pulmonary disease, the increased ventilatory pressure required in this position, is a concern [18]. In this case, isolated lung ventilation was successfully performed using a single-lumen tube and a bronchial blocker. However, manipulation around the trachea can lead to blocker displacement, and relocating the trachea blocker to its original position is difficult for the anesthesiologist to perform in a prone patient. Therefore, we recommend that it may be better to use a double-lumen tube to manage intraoperative ventilation in these patients [19].

## 4 Conclusion

Although mediastinal bronchogenic cysts are encountered relatively often, they are rarely diaphragmatic in origin. As imaging has low diagnostic reliability for these cysts, surgical excision is the most definitive diagnostic and therapeutic modality. We suggest that thoracoscopic surgery with the patient in the prone position may be an optimal approach for the excision of bronchogenic cysts in the posterior mediastinum and should be implemented, depending on the location of the lesion, the patient’s general condition, and the skill of the operator.

## Conflict of interest

The authors declare that they have no conflict of interest.

## Funding

No source of funding to be declared.

## Ethical approval

Ethical approval was not required and patient identifying knowledge was not present in the report.

## Consent

Written informed consent was obtained from the patient for publication of this report and accompanying images.

## Author contribution

Yoshihiro Ota: Drafted the manuscript.

Takafumi Watanabe, Kosuke Takahashi, Takeshi Suda and Shingo Tachibana: Managed the patient.

Jun Matsubayashi: Diagnosed the pathological findings.

Yuichi Nagakawa, Yoshiaki Osaka and Kenji Katsumata: Supervised the writing of the manuscript.

Akihiko Tsuchida: Approved the final manuscript.

## Registration of research studies

Our study does not require registration.

## Guarantor

Akihiko Tsuchida.

## Provenance and peer review

Not commissioned, externally peer-reviewed.
